# Rapid meropenem/vaborbactam NP test for detecting susceptibility/resistance in Enterobacterales

**DOI:** 10.1093/jac/dkad224

**Published:** 2023-08-16

**Authors:** Patrice Nordmann, Auriane Kerbol, Maxime Bouvier, Mustafa Sadek, Laurent Poirel, Otávio Hallal Ferreira Raro

**Affiliations:** Medical and Molecular Microbiology, Faculty of Science and Medicine, University of Fribourg, Fribourg, Switzerland; Swiss National Reference Center for Emerging Antibiotic Resistance (NARA), University of Fribourg, Fribourg, Switzerland; Institute for Microbiology, Lausanne University Hospital and University of Lausanne, Lausanne, Switzerland; Swiss National Reference Center for Emerging Antibiotic Resistance (NARA), University of Fribourg, Fribourg, Switzerland; Swiss National Reference Center for Emerging Antibiotic Resistance (NARA), University of Fribourg, Fribourg, Switzerland; Medical and Molecular Microbiology, Faculty of Science and Medicine, University of Fribourg, Fribourg, Switzerland; Department of Food Hygiene and Control, Faculty of Veterinary Medicine, South Valley University, Qena, Egypt; Medical and Molecular Microbiology, Faculty of Science and Medicine, University of Fribourg, Fribourg, Switzerland; Swiss National Reference Center for Emerging Antibiotic Resistance (NARA), University of Fribourg, Fribourg, Switzerland; Medical and Molecular Microbiology, Faculty of Science and Medicine, University of Fribourg, Fribourg, Switzerland

## Abstract

**Background:**

The treatment options for infections caused by carbapenem-resistant Enterobacterales (CRE) are extremely scarce nowadays and the development of new antibiotics does not follow the exponential increase in the dissemination of carbapenem resistance determinants worldwide. Meropenem/vaborbactam was recently approved for clinical use and it has been indicated for treating several infections. Although relatively rare, meropenem/vaborbactam resistance has already been reported in Enterobacterales and its early detection could be a valuable tool for faster clinical decision-making.

**Objectives:**

To develop a rapid test, namely the Rapid MEV NP, for the identification of meropenem/vaborbactam resistance in Enterobacterales.

**Methods:**

The Rapid MEV NP test is based on detection of glucose metabolization occurring upon bacterial growth in the presence of meropenem/vaborbactam at a concentration of 16/8 mg/L. Bacterial growth is detectable by a colour change of phenol red (from red to yellow) subsequent of the acidification of the medium upon bacterial growth. A total of 75 Enterobacterales isolates were randomly selected for evaluating the performance of the Rapid MEV NP test.

**Results:**

The test showed 97.2% sensitivity and 93.8% specificity when compared with the reference method. The results are obtained after 3 h of incubation at 35°C ± 2°C, which is a gain of time of at least 15 h (one day in practice) compared with currently used antimicrobial susceptibility testing including broth microdilution methods.

**Conclusions:**

The Rapid MEV NP test, easy to perform and to interpret, showed remarkable performance while providing fast results, and is therefore suitable for implementation in routine clinical microbiology laboratories.

## Introduction

Carbapenem-resistant Enterobacterales (CRE) are a significant threat for public health systems worldwide. CRE infections are strongly associated with longer hospitalization, treatment failure and high mortality rate.^1^ The spread of genes encoding carbapenem resistance such as the carbapenemases *bla*_KPC_, *bla*_NDM_, *bla*_VIM_, *bla*_IMP_ and *bla*_OXA-48-like_ has been increasing exponentially during the last decade due to the exchange of successful plasmids of different incompatibility families (e.g. IncF, IncL/M, IncN) harbouring these resistance genes.^2^

The treatment options for infections caused by carbapenemase-producing Enterobacterales (CPE) are extremely limited nowadays. In recent years, the FDA and the EMA approved the β-lactam/β-lactamase inhibitor combinations ceftazidime/avibactam, imipenem/relebactam and meropenem/vaborbactam for clinical use.^3–8^ Meropenem/vaborbactam has strong activity against Ambler class A carbapenemases and class C serine β-lactamases, such as KPC and CMY, respectively.^9,10^

Meropenem/vaborbactam has been indicated to treat complicated urinary tract infections (cUTIs), bacteraemia, abdominal infections, hospital-acquired pneumonia, and also for infections caused by Gram-negative bacteria when other treatments are not effective.^5,6^ Moreover, meropenem/vaborbactam has been preferred over ceftazidime/avibactam because of the increasing reports of acquired resistance to ceftazidime/avibactam observed among Enterobacterales (producing distinct KPC variants or CMY-185).^11–14^

However, although being rarely reported, meropenem/vaborbactam resistance has already been observed in Enterobacterales.^11,15,16^ Among KPC producers, meropenem/vaborbactam resistance has been associated with truncation in OmpK35 and insertion of glycine and aspartic acid within the OmpK36 protein at positions 134–135 (GD134–135).^15,16^ Resistance to meropenem/vaborbactam has been also associated with overexpression of *bla*_KPC-2_ and *bla*_KPC-3_ genes.^17,18^ Therefore, acquired resistance to meropenem/vaborbactam may be resulting from diverse resistance mechanisms. Moreover, vaborbactam does not inhibit either class D (OXA-48) or class B (NDM-1 and VIM-1) MBLs.^9^

The standard reference technique for determining susceptibility to meropenem/vaborbactam is the broth microdilution (BMD), a technique which is rather time-consuming. Other antimicrobial susceptibility techniques such as disc diffusion methods (30 µg discs, Thermo Fisher Scientific, Waltham, USA), ETEST MIC strips (bioMérieux, Marcy-l’Étoile, France), and Sensititre BMD panel MEV (Thermo Fisher Scientific, Waltham, USA) can be used as an alternative but they still require at least 18 h to obtain the results. Currently, the FDA through CLSI (susceptible ≤4 mg/L; intermediate: 8 mg/L; resistant >16 mg/L)^19^ and EUCAST (susceptible ≤8 mg/L; resistant >8 mg/L)^20^ have different breakpoints for interpreting meropenem/vaborbactam susceptibility results for Enterobacterales.

Consequently, the development of a rapid, accurate and reliable test for the detection of meropenem/vaborbactam resistance in Enterobacterales would be a valuable tool for helping clinicians to have faster decision-making for patient treatment. The Rapid MEV NP test was therefore designed for the detection of meropenem/vaborbactam resistance in MDR Enterobacterales, mainly KPC producers. It is basically a glucose metabolization-based test allowing detection of bacterial growth in the presence of a given concentration of meropenem/vaborbactam.

## Methods

### Bacterial strains and antimicrobial susceptibility testing

A total of 84 clinical Enterobacterales strains were randomly selected from the collection of the Swiss National Reference Center of Emerging Antibiotic Resistance (NARA). The Enterobacterales species included *Citrobacter freundii* (3.6%, 3/84), *Enterobacter cloacae* (9.5%, 8/84), *Escherichia coli* (33.3%, 28/84), *Klebsiella aerogenes* (1.2%, 1/84), *Klebsiella pneumoniae* (46.4%, 39/84), *Klebsiella oxytoca* (3.6%, 3/84), *Proteus mirabilis* (1.2%, 1/84) and *Providencia stuartii* (1.2%, 1/84). The carbapenemase content of all strains had been previously characterized (Table 1).

**Table 1.** Rapid MEV test for detection of meropenem/vaborbactam susceptibility testing in Enterobacterales

Species	β-Lactamase	BMD MIC (mg/L)							Phenotype	Rapid MEV test	
		CAZ	CZA	ETP	IPM	IPR	MEM	MEV		Result	Discrepancies versus meropenem/vaborbactam BMD
*E. coli* ATCC 25922	—	≤0.25	≤0.25	≤0.25	≤0.25	≤0.25	≤0.25	≤0.25	S	Negative	—
*E. coli*	CTX-M-1	**>128**	4	**8**	≤0.25	≤0.25	0.5	≤0.25	S	Negative	—
*E. coli*	CTX-M-1	**>128**	8	**128**	4	≤ 0.25	4	0.5	S	Negative	—
*E. coli*	KPC-2	**32**	≤0.25	0.5	2	0.125	0.5	≤0.25	S	Negative	—
*E. coli*	KPC-2	**8**	≤0.25	**8**	2	0.125	4	≤0.25	S	Negative	—
*E. coli*	KPC-2	4	≤0.25	≤0.25	4	0.125	≤0.25	≤0.25	S	Negative	—
*E. coli*	KPC-2	2	≤0.25	≤0.25	2	0.125	0.5	≤0.25	S	Negative	—
*E. coli*	OXA-48	4	≤0.25	**1**	2	2	0.5	≤0.25	S	Negative	—
*E. coli*	OXA-48	2	≤0.25	**1**	0.5	0.25	0.25	≤0.25	S	Negative	—
*E. coli*	OXA-48	1	≤0.25	**1**	1	0.5	0.25	≤0.25	S	Negative	—
*E. coli*	OXA-48	≤0.25	≤0.25	0.5	2	1	≤0.25	0.25	S	Negative	—
*E. coli*	OXA-48	2	≤0.25	**2**	2	2	0.25	0.5	S	Negative	—
*E. coli*	OXA-48	≤ 0.25	≤0.25	**1**	1	1	0.5	0.5	S	Negative	—
*E. coli*	OXA-244	2	≤0.25	**1**	1	0.5	≤0.25	≤0.25	S	Negative	—
*E. coli*	OXA-244	**8**	0.5	**2**	1	1	0.5	≤0.25	S	Negative	—
*K. pneumoniae*	—	**>128**	4	**64**	0.5	≤0.25	0.5	≤0.25	S	Negative	—
*K. pneumoniae*	—	**64**	0.5	**2**	≤0.25	≤0.25	≤0.25	≤0.25	S	Negative	—
*K. pneumoniae*	—	**>128**	8	**2**	≤0.25	≤0.25	≤0.25	≤0.25	S	Negative	—
*K. pneumoniae*	—	**64**	**64**	**>128**	2	≤0.25	8	4	S	Negative	—
*K. pneumoniae*	CTX-M-1	**128**	2	**16**	≤0.25	≤0.25	1	≤0.25	S	Negative	—
*K. pneumoniae*	CTX-M-1	**>128**	8	**16**	≤0.25	≤0.25	≤0.25	≤0.25	S	Negative	—
*K. pneumoniae*	SHV-12	**>128**	**64**	**128**	1	≤0.25	4	2	S	Negative	—
*K. pneumoniae*	KPC-2	**128**	1	**>128**	**128**	0.5	**>128**	1	S	Negative	—
*K. pneumoniae*	KPC-2	**64**	1	**>128**	**128**	0.25	**128**	1	S	Negative	—
*K. pneumoniae*	KPC-2	**64**	2	**>128**	**64**	0.125	**128**	2	S	Negative	—
*K. pneumoniae*	KPC-2	**64**	1	**>128**	**64**	0.25	**128**	8	S	Negative	—
*K. pneumoniae*	KPC-3	**>128**	2	**>128**	**128**	0.125	**128**	1	S	Negative	—
*K. pneumoniae*	KPC-3	**>128**	2	**>128**	**64**	0,06	**128**	0.5	S	Negative	—
*K. pneumoniae*	KPC-3	**>128**	1	**64**	**16**	0.5	**32**	≤0.25	S	Negative	—
*K. pneumoniae*	KPC-3	**>128**	2	**64**	**8**	≤0.25	**32**	≤0.25	S	Negative	—
*K. pneumoniae*	KPC-41	**>128**	**128**	**4**	2	0.25	1	≤0.25	S	Negative	—
*K. pneumoniae*	KPC-46	**>128**	**64**	**8**	0.5	0.5	2	1	S	Negative	—
*K. pneumoniae*	KPC-50	**>128**	**>128**	**16**	**16**	0.5	8	≤0.25	S	Negative	—
*K. pneumoniae*	OXA-48	**128**	0.5	**128**	**8**	**8**	**32**	**16**	R	Negative	VME
*K. pneumoniae*	OXA-181	≤0.25	≤0.25	0.5	0.5	0.5	≤0.25	≤0.25	S	Negative	—
*K. pneumoniae*	VIM-1	**>128**	**>128**	**2**	**8**	**8**	2	1	S	Negative	—
*E. cloacae*	IMI-1	0.5	≤0.25	**128**	**256**	**32**	**64**	≤0.25	S	Negative	—
*E. cloacae*	VIM-1	**>128**	**>128**	**2**	**8**	**8**	1	1	S	Negative	—
*C. freundii*	CTX-M-1	≤0.25	≤0.25	≤0.25	1	≤0.25	≤0.25	0.25	S	Negative	—
*C. freundii*	KPC-2	**32**	4	**64**	**32**	0.5	**16**	≤0.25	S	Negative	—
*C. freundii*	OXA-181	**>128**	1	**32**	2	2	4	4	S	Negative	—
*K. oxytoca*	KPC-3	**64**	≤0.25	**16**	**8**	0.5	8	≤0.25	S	Negative	—
*K. oxytoca*	OXA-48	≤0.25	≤0.25	**1**	2	0.5	0.5	0.5	S	Negative	—
*K. oxytoca*	OXA-48	≤0.25	≤0.25	**1**	4	2	0.5	0.5	S	Negative	—
*K. aerogenes*	KPC-3	**>128**	1	**64**	**16**	2	**32**	≤0.25	S	Negative	—
*P. mirabilis*	OXA-23	≤0.25	≤0.25	0.25	**8**	**8**	0.5	0.5	S	Negative	—
*P. stuartii*	NDM-1	**>128**	**>128**	≤0.25	**32**	**32**	0.5	1	S	Negative	—
*E. coli*	NDM-5	**>128**	**>128**	**16**	**8**	**8**	**32**	**16**	R	Positive	—
*E. coli*	NDM-5	**>128**	**>128**	**32**	**16**	**16**	**32**	**16**	R	Positive	—
*E. coli*	NDM-5	**>128**	**>128**	**64**	**8**	**8**	**32**	**16**	R	Positive	—
*E. coli*	NDM-5	**>128**	**>128**	**64**	**32**	**32**	**32**	**16**	R	Positive	—
*E. coli*	NDM-5	**>128**	**>128**	**32**	**8**	**4**	**32**	**16**	R	Positive	—
*E. coli*	NDM-5	**>128**	**>128**	**64**	**16**	**16**	**64**	**32**	R	Positive	—
*E. coli*	NDM-5	**>128**	**>128**	**128**	**16**	**16**	**64**	**64**	R	Positive	—
*E. coli*	NDM-5	**>128**	**>128**	**64**	**16**	**16**	**64**	**64**	R	Positive	—
*E. coli*	NDM-5	**>128**	**>128**	**128**	**32**	**32**	**>128**	**128**	R	Positive	—
*E. coli*	NDM-5	**>128**	**>128**	**>128**	**16**	**16**	**>128**	**128**	R	Positive	—
*E. coli*	NDM-5	**>128**	**>128**	**>128**	**16**	**16**	**128**	**128**	R	Positive	—
*E. coli*	NDM-5	**>128**	**>128**	**>128**	**16**	**16**	**128**	**>128**	R	Positive	—
*E. coli*	NDM-5 + OXA-48	**>128**	**>128**	**64**	**16**	**16**	**64**	**64**	R	Positive	—
*E. coli*	NDM-5 + OXA-181	**>128**	**>128**	**64**	**8**	**8**	**32**	**32**	R	Positive	—
*K. pneumoniae*	KPC-2	**128**	4	**>128**	**64**	0.5	**>128**	**16**	R	Positive	—
*K. pneumoniae*	KPC-2	**>128**	4	**>128**	**128**	1	**>128**	**32**	R	Positive	—
*K. pneumoniae*	KPC-3	**>128**	**16**	**>128**	**>128**	2	**>128**	**16**	R	Positive	—
*K. pneumoniae*	NDM-1	**>128**	**>128**	**8**	**8**	**8**	8	8	S	Positive	ME
*K. pneumoniae*	NDM-1	**>128**	**>128**	**16**	**16**	**16**	8	**16**	R	Positive	—
*K. pneumoniae*	NDM-1	**>128**	**>128**	**64**	**8**	**8**	**16**	**16**	R	Positive	—
*K. pneumoniae*	NDM-1	**>128**	**>128**	**32**	**8**	**8**	**16**	**32**	R	Positive	—
*K. pneumoniae*	NDM-1	**>128**	**>128**	**64**	**16**	**8**	**32**	**32**	R	Positive	—
*K. pneumoniae*	NDM-1	**>128**	**>128**	**64**	**32**	**32**	**64**	**32**	R	Positive	—
*K. pneumoniae*	NDM-1	**>128**	**>128**	**>128**	**32**	**32**	**>128**	**128**	R	Positive	—
*K. pneumoniae*	NDM-4	**>128**	**>128**	**64**	**16**	**32**	**64**	**32**	R	Positive	—
*K. pneumoniae*	NDM-4 + OXA-181	**>128**	**>128**	**64**	**16**	**16**	**64**	**64**	R	Positive	—
*K. pneumoniae*	NDM-5	**>128**	**>128**	**32**	**8**	**16**	**64**	**64**	R	Positive	—
*K. pneumoniae*	NDM-5 + OXA-181	**>128**	**>128**	**>128**	**128**	**64**	**>128**	**128**	R	Positive	—
*K. pneumoniae*	NDM-5 + OXA-181	**>128**	**>128**	**>128**	**64**	**64**	**128**	**128**	R	Positive	—
*K. pneumoniae*	OXA-48	**>128**	≤0.25	**>128**	**128**	**64**	**>128**	**128**	R	Positive	—
*K. pneumoniae*	OXA-48	**16**	1	**64**	**8**	**8**	**32**	**16**	R	Positive	—
*K. pneumoniae*	OXA-232	1	0.5	**128**	1	1	**16**	**16**	R	Positive	—
*E. cloacae*	NDM-1	**>128**	**>128**	**4**	4	**4**	4	2	S	Positive	ME
*E. cloacae*	NDM-1	**>128**	**>128**	**16**	**8**	**8**	8	8	S	Positive	ME
*E. cloacae*	NDM-1	**>128**	**>128**	**>128**	**32**	**64**	**32**	**32**	R	Positive	—
*E. cloacae*	NDM-1 + OXA-48	**>128**	**>128**	**128**	**32**	**32**	**32**	**32**	R	Positive	—
*E. cloacae*	NDM-5	**>128**	**>128**	**64**	**16**	**16**	**32**	**32**	R	Positive	—
*E. cloacae*	NDM-7	**>128**	**>128**	**32**	**16**	**32**	**32**	**16**	R	Positive	—

CAZ, ceftazidime; CZA, ceftazidime/avibactam; ETP, ertapenem; IPM, imipenem; IPR, imipenem/relebactam; MEM, meropenem; MEV, meropenem/vaborbactam; S, susceptible; R, resistant; —, no discrepancies observed. For BMD results, bold type indicates resistant, underlining indicates intermediate and normal script indicates susceptible.

The BMD reference test was performed to determine the MIC of all the studied strains, following the EUCAST guidelines. Results were interpreted according to the EUCAST breakpoints.^20^ BMD was considered the gold standard when comparing with the results obtained with the Rapid MEV NP test. The reference strain *E. coli* ATCC 25922 was used as quality control for both techniques.

### Rapid MEV NP test

Several different parameters were tested to obtain the optimal conditions for the test, as described below. We tested different pH values for the culture medium (6.7 and 7.3), different bacterial inoculum (0.5 McFarland’s scale, 1/5 and 1/10 of the 0.5 McFarland’s scale), different meropenem/vaborbactam concentrations (8/4, 8/8, 16/4, 16/8, 16/16, 16/32, 64/8, 64/16 and 64/32 mg/L), and different incubation times (2, 3 and 4 h).

To prepare 250 mL of Rapid MEV NP solution, 0.0125 g of the pH indicator phenol red (Sigma–Aldrich, St. Louis, USA), 6.25 g of CAMHB (Bio-Rad, Marnes-la-Coquette, France) and 200 mL of distilled water were added in a glass bottle. Twenty-five millilitres of 0.01 M ZnSO_4_ (Roth, Karlsruhe, Germany) was then added. The pH solution was adjusted to 7.3 by adding drops of 1 mol/L NaOH. This solution was then autoclaved at 121°C for 15 min. After cooling down the solution to room temperature, 25 mL of 10% anhydrous filter-sterilized D(+)-glucose (Roth, Karlsruhe, Germany) was added. Final concentrations of the Rapid MEV NP solution therefore corresponded to 2.5% CAMHB powder, 0.005% phenol red, 0.1 mM ZnSO_4_ and 1% D(+)-glucose. The Rapid MEV NP solution can be kept at 4°C for 1 week or at −20°C for at least 6 months.

Meropenem (Hui Chem, Shanghai, China) and vaborbactam (MedChemExpress, New Jersey, USA) compounds were used to prepare a final concentration at 16 and 8 mg/L, respectively, after having tested different concentration of both molecules.

### Bacterial inoculum

Fresh overnight cultures were grown on UriSelect 4 (Bio-Rad) or Mueller–Hinton agar plates (Bio-Rad). After that step, a standardized bacterial inoculum of 0.5 McFarland scale was prepared by adding bacterial colonies into 5 mL of sterile NaCl (0.85%). Bacterial suspensions were inoculated in a range from 15 to 1 h after preparation, following the EUCAST guidelines recommendation for susceptibility testing.^20^

### Tray inoculation

A 96-well polystyrene microplate (round base, with lid, sterile; Sarstedt, Germany) was used to inoculate the 0.5 McFarland bacterial suspension in two independent wells, with and without meropenem/vaborbactam. The steps to perform the Rapid MEV NP test were: (i) 100 µL of meropenem/vaborbactam-free Rapid MEV NP solution was added to wells A1–A4; (ii) 50 µL of meropenem at a concentration of 48 mg/L was added to wells B1–B4; (iii) 50 µL of vaborbactam at a concentration of 24 mg/L was added to wells B1–B4; (iv) 50 µL (0.5 McFarland) of *E. coli* ATCC 25922 (negative control) was added to wells A1 and B1; (v) 50 µL of a meropenem/vaborbactam-resistant isolate (positive control) was added to wells A2 and B2; (vi) 50 µL of a tested isolate was added to wells A3 and B3; and (vii) 50 µL of NaCl 0.85% was added to wells A4 and B4.

Consequently, each well had a final volume of 150 µL and the final concentration of meropenem/vaborbactam was 16/8 mg/L. Of note, after preparing the microplate for the test and before inoculating the bacterial suspensions, the Rapid MEV NP solution was pre-warmed for 15–30 min at 37°C before use to prevent growth delay and therefore a delayed colour change.

### Tray incubation and reading

The test was incubated for up to 3 h at 35 ± 2°C in ambient air, with the lid, and without agitation. The tray was not sealed to enable carbohydrate metabolism through oxygen consumption.

Reading was performed visually by checking the tray for no spontaneous colour change after 30 min, and then every 1 h until reaching 3 h of incubation. Interpretation of the results was performed as follows: an isolate was considered as positive (resistant) when it grew in the presence of meropenem/vaborbactam, and as negative (susceptible) when no growth was observed in the presence of meropenem/vaborbactam after 3 h of incubation. The quality control of the test was considered optimal, therefore validating the Rapid MEV NP test, if the following conditions were reached; (i) red-to-yellow colour change observed, confirming the bacterial growth and glucose metabolism for all isolates in wells without meropenem/vaborbactam (A1–A3); (ii) no colour change observed (remaining red) in wells after adding NaCl 0.85%, confirming the absence of contamination (A4 and B4); (iii) red to yellow colour changes observed in the wells where the positive control and the tested isolate were added (A2 and B2; A3 and B3); (iv) no colour change observed for *E. coli* ATCC 25922 in the well with meropenem/vaborbactam (negative control) (B1).

**Figure 1.**
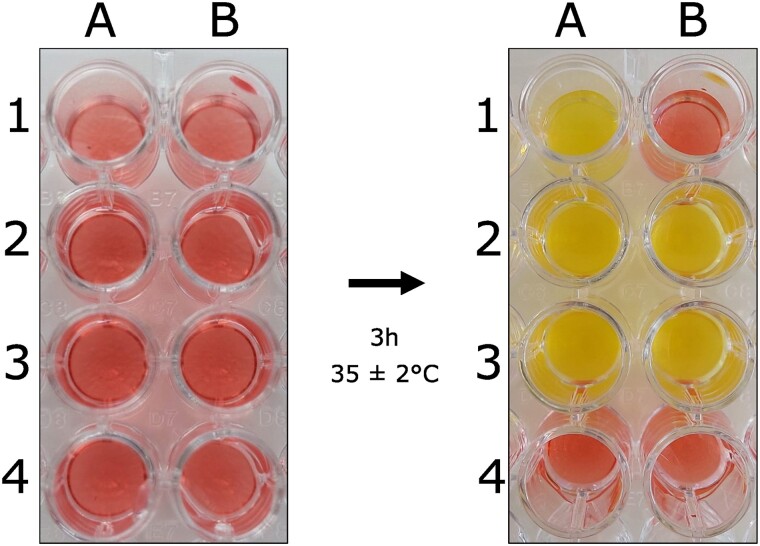
The Rapid MEV NP test. Column A presents the Rapid MEV NP solution free of meropenem/vaborbactam; column B presents the MEV NP solution with meropenem/vaborbactam (16/8 mg/L). Reference strain *E. coli* ATCC 25922 was inoculated in A1 and B1; meropenem/vaborbactam-resistant isolate (positive control) was inoculated in A2 and B2; Tested isolate (resistant to meropenem/vaborbactam) that grew in both absence and presence of meropenem/vaborbactam was inoculated in A3 and B3; and NaCl 0.85% was inoculated in A4 and B4 as control of contamination and possible spontaneous colour change. Bacterial growth is evidenced by a colour change of the medium from red to yellow.

The test was considered positive when the isolate resistant to meropenem/vaborbactam grew in the wells in the absence and presence of meropenem/vaborbactam, with an identical red-to-yellow colour change in both wells (wells A2–A3 and B2–B3). The result was interpreted as negative when the well corresponding to the tested isolate remained red, or alternatively showed a slight colour change from red to orange. Figure 1 provides a comprehensive illustration of the visual interpretation of the Rapid MEV NP test.

### Data analysis

Results obtained with the Rapid MEV NP test were compared with those obtained with the BMD standard reference method. Discrepancies between these two tests were determined and classified as very major errors (VMEs) and major errors (MEs) as previously described.^21^ MEs were defined as isolates giving a positive result with the Rapid MEV NP test but being susceptible to meropenem/vaborbactam by BMD (false-positive result). VMEs corresponded to isolates giving a negative result with the Rapid MEV NP test but being resistant to meropenem/vaborbactam by BMD (false-negative result). Sensitivity, specificity, accuracy/categorical agreement, and precision parameters were also calculated to evaluate the performance of the test proposed. Results were blindly read and interpreted independently by two laboratory members.

## Results

The tested strain collection included 84 randomly selected Enterobacterales including KPC producers [*n* = 21 (25%); KPC-2, −3, −41, −46, −50], NDM producers [*n* = 27 (32.1%); NDM-1, −4, −5, −7], VIM-1 producers [*n* = 2 (2.4%)], OXA-23 or OXA-48-like producers [*n* = 17 (20.2%); OXA-23, −48, −181, −232, −244], OXA and NDM co-producers [*n* = 6 (7.1%)], IMI-1 producer [*n* = 1 (1.2%)], CTX-M-1 producers [*n* = 5 (6.0%)], SHV-12 producer [*n* = 1 (1.2%)] and non-β-lactamase producers [*n* = 4 (4.8%)]. Among these isolates, 42.9% (36/84) were resistant to meropenem/vaborbactam (MICs ranging from 16 to >128 mg/L) and 57.1% (48/84) remained susceptible (MICs ranging from ≤0.25 to 8 mg/L) according to the BMD results and interpreted according to the EUCAST guidelines. These results are summarized in Table 1.

Overall, 45 out of the 48 meropenem/vaborbactam-susceptible isolates gave negative results with the Rapid MEV NP test. Two out of the three false-positive isolate results actually showed borderline MIC of meropenem/vaborbactam (8 mg/L), the MIC of the remaining isolate being 2 mg/L; all these three isolates were NDM-1 producers (Table 1). A total of 35 out of the 36 meropenem/vaborbactam-resistant isolates tested gave positive results with the Rapid MEV NP test. Only a single isolate showing an MIC of meropenem/vaborbactam at 16 mg/L and being an OXA-48 producer gave a negative result with the Rapid MEV test (Table 1).

Consequently, the Rapid MEV NP test gave 6.3% (3/48) ME (false positive) and 2.8% (1/36) VME (false negative) results. The Rapid MEV NP test showed strong correlation with the BMD results. It showed 97.2% (95% CI 85.8%–99.5%) sensitivity, 93.8% (95% CI 83.2%–97.9%) specificity, 95.2% accuracy/categorical agreement and 92.1% precision when compared with the BMD standard method. After reading the colour change of the wells at each hour, it was concluded that the optimal reading of the final results should be at 3 h after incubation at 35°C ± 2°C under an ambient atmosphere.

## Discussion

Occurrence of CPE is a great concern to public health due to the associated limited treatment options, high morbidity and mortality, and remarkable ability to rapidly disseminate worldwide.^22^ Despite the efforts made on infection control and antibiotic stewardship practices, the exponentially growing dissemination of CPE remains a critical issue.

Here, we propose a novel, rapid, accurate and reliable test to determine meropenem/vaborbactam susceptibility/resistance in Enterobacterales. The Rapid MEV NP test was able to detect meropenem/vaborbactam resistance within 3 h, being at least 15 h faster than the currently available testing methods such as disc diffusion, ETEST MIC strips, Sensititre BMD panel MEV and the reference gold standard BMD. Taking into account the diversity of mechanisms associated with meropenem/vaborbactam resistance, this test, which is based on a rapid phenotypic technique and therefore fully independent of the nature of the mechanism, if any, is perfectly adapted to the evaluation of meropenem/vaborbactam susceptibility/resistance. On the contrary, immune-chromatographic, biochemical and molecular-based techniques seem therefore not adaptable for such evaluation.

Although very few discrepancies were observed when compared with the BMD (three MEs and a single VME), the Rapid MEV NP test showed significant correlation with the standard technique in terms of sensitivity, specificity, accuracy/categorical agreement and precision parameters. Of note, three out of four discrepancies were identified for isolates presenting borderline MICs (8 and 16 mg/L). A limitation of the present study is that only a limited number of meropenem/vaborbactam-resistant KPC producers were available to be tested, since this profile remains relatively scarce.

Also noteworthy is the consideration of EUCAST breakpoints for meropenem/vaborbactam for the development of this Rapid MEV NP test.^13^ If analysing the results considering the CLSI breakpoints (susceptible, ≤4 mg/L; intermediate, =8 mg/L; resistant, >16 mg/L)^19^ and setting resistant and intermediate isolates categorized as positive for the test, the performance of the test remains remarkable, with only one ME (false positive, 2.1%) and 2 VMEs (false positive, 5.6%, both presenting borderline MICs of 8 and 16 mg/L), 94.9% sensitivity (95% CI 83.1%–98.6%), 97.8% specificity (95% CI 88.4%–99.6%), 96.4% accuracy/categorical agreement and 97.4% precision.

### Conclusions

The Rapid MEV NP test is inexpensive, easy to perform and interpret, and enables rapid detection of meropenem/vaborbactam susceptibility of Enterobacterales. It is therefore suitable for implementation in routine clinical microbiology laboratories. Clinical validation of this rapid test now requires further evaluations in different laboratories and different geographical area with diverse epidemiology.

## Transparency of declarations

None to declare.
